# Review of metastasis to meningiomas with case examples

**DOI:** 10.1016/j.bas.2024.102862

**Published:** 2024-07-06

**Authors:** Magnus Sættem, Terje Sundstrøm, Anna.K.Myrmel Sæle, Rupavathana Mahesparan

**Affiliations:** a Department of Neurosurgery, Haukeland University Hospital, Bergen, Norway; b Department of Biomedicine, University of Bergen, Bergen, Norway; c Department of Clinical Medicine, University of Bergen, Norway; d Department of Pathology, Haukeland University Hospital, Bergen, Norway

**Keywords:** Metastasis, Tumor-to-meningioma metastasis, Prostate, Case report, Meningioma, Adenocarcinoma

## Abstract

**Introduction:**

A tumor-to-tumor metastasis (TTM) is a rare metastatic process where a primary malignant tumor metastasizes to another tumor, most commonly a benign tumor such as a meningioma. Here, we present two recent cases of tumor-to-meningioma metastases (TMM) from our clinical practice and review of recent literature. The primary cancers were prostate and breast cancer, respectively.

**Material and methods:**

We reviewed the electronic medical records of the two patients and conducted a literature review of TTM, focusing on biological mechanisms related to TMM.

**Results:**

Our first patient, a man with a history of stable prostate cancer, underwent resection of two WHO grade 1 meningiomas, and the largest tumor was found to have TMM. Our second patient, a woman with progressive breast cancer, was operated for a WHO grade II meningioma, and the meningioma harbored breast cancer metastases. TMM is a rare occurrence, but breast cancer is a much more frequent cause than prostate cancer and we reviewed 50 cases. Only 15 of cases of TMM from prostate cancer have been described.

**Discussion and conclusion:**

TMM is a rare phenomenon, but it is important to be aware of this as more and more patients live with cancer and meningiomas have a high prevalence, The possibility of TMM may impact not only both the surgical and oncological treatment but also surveillance of incidental meningiomas.

## Highlights

• Tumor-to-tumor metastasis (TTMM) is a rare occurrence where a primary malignant tumor spreads to a benign tumor like a meningioma.
• The report includes two cases of TTMM: one from prostate cancer to a WHO grade I meningioma, and another from breast cancer to a WHO grade II meningioma.
• Distinct markers were found in each case, such as CKAE1/AE3 and PSA in the prostate cancer case, and CKAE1/AE3 and CK7 in the breast cancer case.
• Recognizing TTMM is crucial for patient care, suggesting increased screening for meningiomas in patients with known cancers.
• TTMM affects surgical and oncological treatment strategies, requiring a multidisciplinary approach for accurate diagnosis and management.

## Abbreviations

TTM –: tumor-to-tumor metastasis
TMM –: tumor-to-meningioma metastasis
MRI -: magnetic resonance imaging
PSA -: Prostate Specific Antigen
BBB -: blood-brain barrier
PSMA -: prostate-specific membrane antigen
pMRI -: perfusion magnetic resonance imaging
PET-CT -: positron-emission tomography-CT
WHO –: World Health Organization

## 1 Introduction

Brain metastases are a common clinical problem (Daphu et al., 2013). A tumor-to-tumor metastasis (TTM) is however a rare metastatic process in which a primary malignant tumor metastasizes to another tumor (Li et al., 2023). Virtually any benign or malignant tumor can be a recipient, but meningiomas are the most common intracranial neoplasms to harbor a metastasis (Moody et al., 2012). Since the first case described by Fried in 1930, only about 150 cases of tumor-to-meningioma metastasis (TMM) have been reported (Fried, 1930; Turner et al., 2021a).

Breast cancer is one of the most frequent causes of brain metastases and are also the most common culprits in TMM (Pham et al., 2018). Prostate cancer is the second most common cancer in men, but brain metastases from prostate cancer are exceedingly rare (Rajeswaran et al., 2022). Less than 15 cases of prostate cancer with TMM have previously been described (Turner et al., 2021a).

Knowing that meningiomas are highly prevalent in the population (Van Allen et al., 2014), it is important to be aware of the possibility of TMM, as an increasing number of patients are diagnosed with cancer and patients are living longer with their cancer diagnosis (Van Allen et al., 2014).

Here, we report two cases of TMM, from prostate and breast cancer, respectively. We also provide a brief overview of the literature on TMM, focusing on the biological mechanisms associated with TMM.

## 2 Case reports

### 2.1 Case 1:

An 86-year-old man with a stable and localized prostate cancer was referred to a magnetic resonance imaging (MRI) scan after a period of dizziness and several falls in the home. The patient reported no other symptoms other than remembering dates. A neurological exam revealed no neurological deficits except for an absent biceps reflex in the left arm and unsteadiness. His Prostate Specific Antigen (PSA) levels were stable (0–6,5 μg/L).

The MRI scan showed two extra-axial lesions of different sizes (Fig. 1). The largest (6,2 x 3,1 x 2,8 cm) was located parasagittaly in the right frontal region, and showed contrast enhancement with variable signal intensity, perifocal edema, and mass effect. The smaller lesion had a similar appearance and was located more laterally. The lesions were diagnosed radiologically as probable meningiomas.

**Fig – 1:**
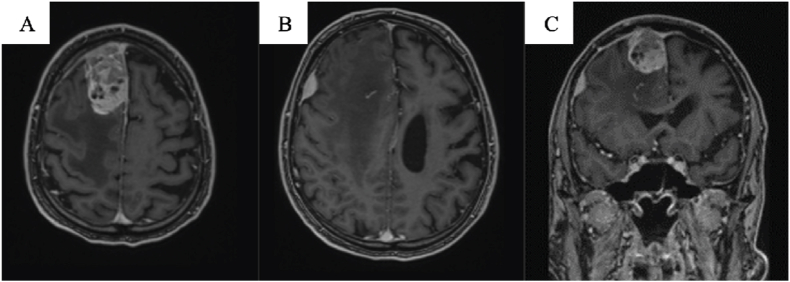
Contrast-enhanced T1-weighted images before surgery. A & B: MRI in axial plane. C: MRI in coronal plane.

A right-sided frontotemporal craniotomy was performed with resection of the two tumors. The patient was in good condition after surgery. A postoperative MRI scan showed persistent edema, but no residual tumor on contrast-enhanced T1-weighted images (Fig. 2).

**Fig – 2:**
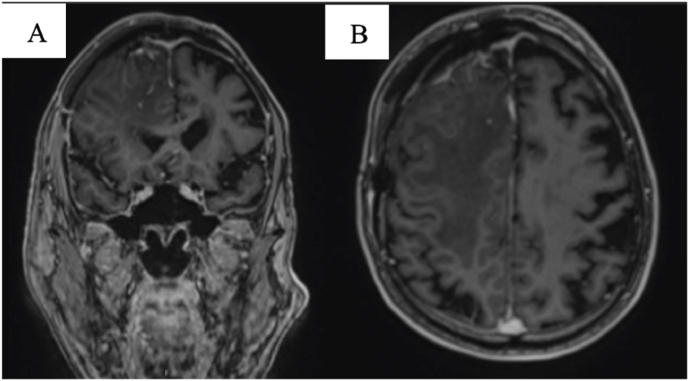
Contrast-enhanced T1-weighted images after surgery. A: MRI in coronal plane. B: MRI in axial plane.

In the histological examination, the smallest tumor had meningothelial appearance with low mitotic activity. The largest tumor showed two distinct types of tissue, where meningothelial tissue was infiltrated by malignant tumor tissue with an epithelial appearance. Immunohistochemistry showed that the epithelial tumor cells were positive for CKAE1/AE3, PSMA, NKX3-1, and PSA. It was negative for EMA, vimentin, and progesterone receptor. The smallest tumor was identified as a meningioma (CNS WHO grade 1), and the largest tumor was identified as a meningioma (CNS WHO grade 1) with TMM from adenocarcinoma, consistent with a primary prostate cancer (Fig. 3). Further investigations revealed signs of metastases from his prostate cancer to the pelvic bone and lymph nodes in the abdomen and pelvis. The patient received further treatment with Zoladex (Gosereline) every third month. He did not receive any further direct treatment of the brain lesions. His latest PSA was 0,1 μg/L (0–6,5), and his latest MRI did not show any signs of tumor relapse.

**Fig – 3:**
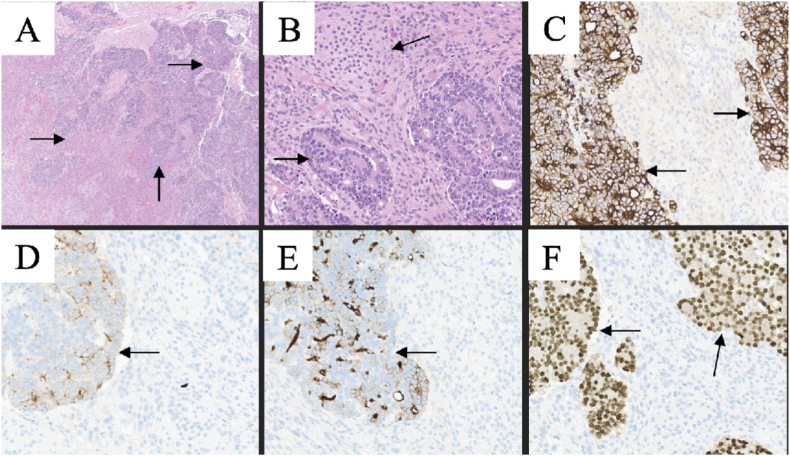
Histology of a meningioma (CNS WHO grade 1) with tumor-to-meningioma metastasis from adenocarcinoma; hematoxylin and eosin staining at low and high magnification (A–B). The adenocarcinoma showed positive immunohistochemical staining for; CKAE1/AE3 (C), PSA (D), PSMA (E), and NKX3-1 (F).

### 2.2 Case 2:

A 54-year-old woman with progressive breast cancer and widespread metastases to the liver, bones, and spine, was referred to an MRI of the brain and spine after she presented with back pain, unsteadiness, morning headaches, and dizziness. The brain MRI showed an extra-axial lesion in the right frontal region with relatively homogenous contrast enhancement (Fig. 4). The lesion measured 4,4 x 4,3 x 4,1 cm and was diagnosed as meningioma.

**Fig – 4:**
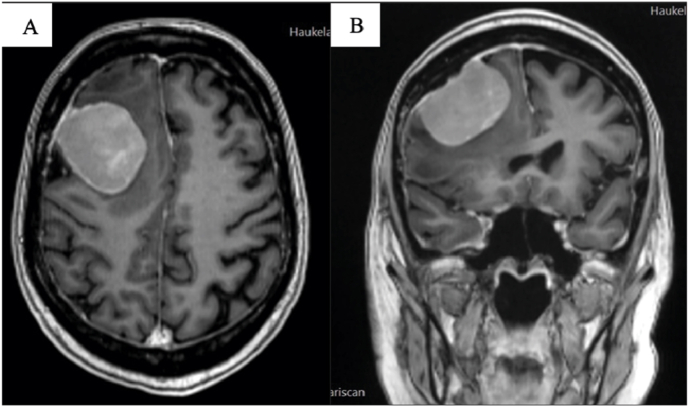
Contrast-enhanced T1-weighted images before surgery. A: MRI in axial plane. B: MRI in coronal plane.

The patient was operated with a frontal craniotomy with *en bloc* removal of the tumor and the adjacent dura (Fig. 5). The tumor had the morphology of a meningioma, and this was confirmed by intraoperative frozen section.

**Fig – 5:**
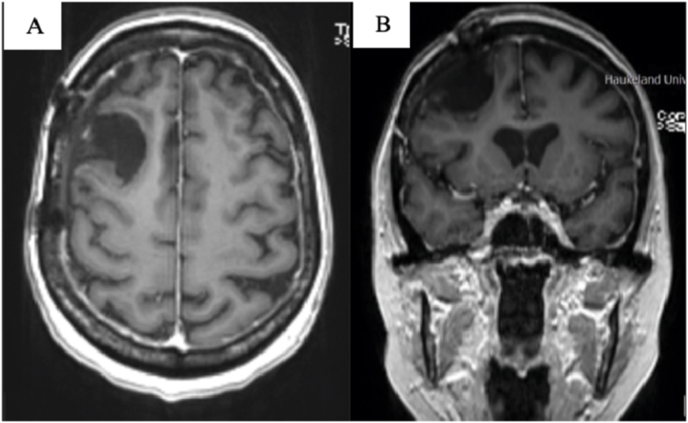
Contrast-enhanced T1-weighted images after surgery. A: MRI in axial plane. B: MRI in coronal plane.

The histological examination showed a meningothelial tumor with increased mitotic activity and brain invasion, and the tumor was identified as an atypical meningioma (CNS WHO grade 2). Single files and groups of cells with an atypical epithelial appearance with high mitotic activity were identified within the tumor. The immunohistochemistry showed that these cells were positive for CKAE1/AE3 and CK7. The tumor was diagnosed as meningioma (WHO grade 2) with TMM from breast carcinoma (lobular carcinoma of pleomorphic subtype). She did not get any further treatment for cerebral metastasis (Fig. 6). The patient was on Paclitaxel (chemotherapy) until she got admitted to the hospital one month later with fever and a high CRP. Antibiotics were started for an assumed lung infection and pleural drainage was done several times, but she further deteriorated with kidney failure and succumbed to her disease. An autopsy revealed widespread metastases to all lung lobes.

**Fig – 6:**
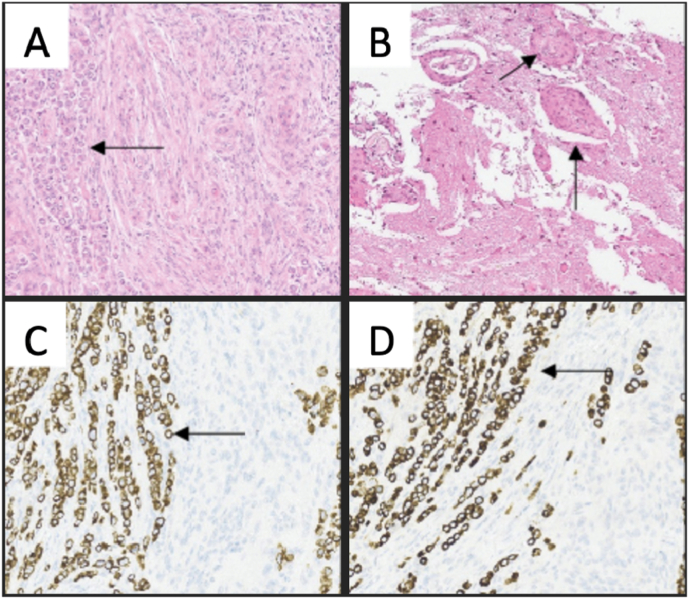
Histology of an atypical meningioma (CNS WHO grade 2) with tumor-to-meningioma metastasis from groups and single files of carcinoma cells (A). Brain invasion of meningothelial tumor tissue (B). The carcinoma was immunoreactive for CKAE1/AE3 (C), and CK7 (D).

## 3 Discussion

Breast cancer is a frequent cause of brain metastases, whereas brain metastases from prostate cancer are extremely rare (Al-Salihi et al., 2021). The most common sources of TMM are breast and lung carcinomas (Sayegh et al., 2015). A meta-analysis from 2021 on TMM included 124 articles with 152 cases of patients with TMM (Turner et al., 2021a). The cancer origin was reported for 149 cases, of which 50 were from breast cancer. Several reviews of TMM from breast cancer have been published (Turner et al., 2021a; Caroli et al., 2006; Erdogan et al., 2014; Rzehak et al., 2022). To the best of our knowledge, fewer than 15 cases of TMM from prostate cancer have been described (Moody et al., 2012; Turner et al., 2021a; Wilson et al., 2023; Bernstein et al., 1983; Chambers et al., 1980; Cluroe, 2006; Döring, 1975; Mitchell et al., 2011; Neville et al., 2017; Pugsley et al., 2009)

Pamphlett's criteria are essential for diagnosing TTMM: the metastatic focus must be at least partially enclosed by a rim of benign host tumor tissue, and the existence of the metastasizing primary carcinoma must be proven (Pamphlett, 1984). Both our cases met these criteria, illustrating the variability in primary cancers leading to TTMM.

Various pathophysiological mechanisms have been implicated in TTM. Three conditions have been proposed as necessary for the establishment of TTM in the recipient tumor: (Daphu et al., 2013) it must be hypervascular, allowing it to be affected by hematogenous metastasis, (Li et al., 2023) it must be richly nourished, allowing the growth of donor tumor cells; and 3) it should be characterized by slow growth (Minezaki et al., 2022). Meningiomas are often highly vascularized tumors (Turner et al., 2021a), that typically grow slowly (Hashimoto et al., 2012; Lyndon et al., 2019). Moreover, meningiomas usually have their main blood supply from the external carotid artery and are thus not protected by the blood-brain barrier (BBB) (Ansari et al., 2020; Long, 1973). Meningiomas have a high prevalence in the population, likely making them the most common recipient tumors. In contrast, TTM has been rarely described in benign tumors, such as pituitary adenomas and vestibular schwannomas (Papadakis et al., 2021).

High vascularity alone cannot account for the process of TTM, as evidenced by the rarity of TTM in glioblastomas, despite their highly vascular nature (Galev et al., 2023). This indicates that additional factors beyond vascular supply must play a role in the occurrence of TTM in meningiomas. It has been pointed out that the ability of various tissues to accept metastases not only depends on the vascular anatomy of the tissue, but also on its metabolic and biological properties (Smith et al., 1981). A well-nourished and slowly growing lesion such as a meningioma probably provides a more favorable microenvironment for metastasis compared to a rapidly growing lesion, such as a glioma (Döring, 1975). The high collagen and lipid content of meningiomas has also been postulated to provide a “fertile soil” for the seeding of malignant cells (Lanotte et al., 2009). The lack of a host immune response within meningiomas makes the tumor an immune haven for metastasis (Li et al., 2023). Finally, overexpression of oncogenes in meningiomas and metastatic carcinomas can also contribute to the simultaneous occurrence (Neville et al., 2017).

Meningiomas with metastases are more likely to express hormone receptors and adhesion molecules (Johnson, 2022). Cell adhesion molecules such as ICAM, B1 integrin, PECAM-1, P-selectin, CXCL12, and SDF-1 have also been proposed as part of the mechanism underlying breast carcinoma metastasis, and ICAM expression in meningiomas may also facilitate adhesion of metastases to meningioma blood vessels (Johnson, 2022; Mogere et al., 2024). Our immunohistochemical analysis found markers CKAE1/AE3 and PSA in the prostate cancer case, and CKAE1/AE3 and CK7 in the breast cancer case, significance of these in TMM is yet to be clarified.

The metastatic ability of tumor cells is generally known to be dependent on the interaction between tumor cells and the microenvironment of the target organ (Ungefroren et al., 2011). Mucin-16 (cancer antigen 125), a protein that is found in metastatic adenocarcinoma, has a high affinity for mesothelin, which has an increased expression in meningiomas (Syed et al., 2018). This affinity between metastatic tumor cells and the target organ is thought to play a role in invasion and metastasis of the donor cells to the recipient (Minezaki et al., 2022). Our patients had two different subtypes of meningioma (grade 1 and II), but according to a literature review of metastases to meningiomas, there is no increased risk with different meningioma subtypes (Johnson, 2022).

A study from 2022 reported that prostate-specific membrane antigen (PSA), expressed in the prostate epithelium that is upregulated in prostate adenocarcinoma, was expressed in 98,9% of meningioma specimens within their endothelial cells (Tubre et al., 2022).

Studies have identified an association between breast carcinoma and meningioma, both of which occur twice as frequently in women and the correlation suggests a hormonal component, potentially involving progesterone receptors (Blankenstein et al., 2000; Hsu et al., 1997). Both breast carcinoma and meningioma have been linked to hormone receptors in their genesis and progression. Approximately 88% of meningiomas express progesterone receptors, but only 30% express estrogen receptors (Degeneffe et al., 2023). Breast cancer is more prevalent in women with meningiomas than in the general female population (Degeneffe et al., 2023). The expression of cellular adhesion molecules like E-cadherin may explain the tendency for breast cancers to metastasize to meningiomas (Aghi et al., 2005). This suggests that women with meningiomas should be more intensively screened for breast cancer, and vice versa. Consequently, our department has increased follow-up frequency for female patients with small meningiomas and known breast cancers.

In over one-third of cases in a systematic review, TMM was the first sign of previously unknown cancer (Turner et al., 2021a). Detecting TMM in patients without a history of metastatic disease remains challenging. All reported TMM cases for prostate cancer involved relatively older patients. The incidence of meningiomas, which peaks at a median age of 66, increases with age (Ogasawara et al., 2021). With rising cancer rates and an aging population, this phenomenon may become more common, as metastatic adenocarcinoma has a high affinity for meningiomas. Since histopathological examination is currently the only definitive diagnostic method, surgery is required to detect these lesions, as clinical and radiological findings are neither specific nor sensitive for TMM (Neville et al., 2017).

The wait-and-watch approach is often used in patients with newly diagnosed incidental meningiomas as most incidental meningiomas are small, demonstrate indolent behavior during follow-up, and do not require intervention (Islim et al., 2023). In patients with diagnosed cancer clinicians should perhaps not underestimate small meningiomas, as these could be potential metastasis targets. Meningioma patients with a known primary cancer should undergo more frequent screening (Turner et al., 2021b). Aggressive growth of the meningioma, possibly with increasing neurological deteriorating, could indicate the presence of a TMM in a patient with known cancer. In 95% of the cases of TMM described in the literature, the lesion was symptomatic at presentation (Turner et al., 2021a). However, a correct preoperative diagnosis is still a major challenge. Patients with known cancer receiving surgery for a meningioma may benefit from a more extensive resection (Simpson grade 1) with complete removal including resection of the underlying bone and associated dura.

Moody and Caroli suggest that atypical changes of signal on routine MRI may indicate the coexistence of two different tumors (Moody et al., 2012; Caroli et al., 2006). This was the case of our patient with prostate cancer. More use of physiology-based neuroimaging modalities, such as perfusion MRI (pMRI), positron-emission tomography-CT (PET-CT) and magnetic resonance spectroscopy may result in better diagnostic yield for this condition as this can assess the metabolic pattern of a tumor (Neville et al., 2017). Jun et al. demonstrated how pMRI could identify regions of hemodynamic differences between two unique tissue types that were not apparent on conventional MRI (Jun et al., 2006). As the authors suggest, more rigorous and systematic validation is necessary by correlating pMRI-derived physiological data with histopathology and outcome before pMRI is incorporated into standard patient care.

Further research is required to assess the impact of TMM on treatment decisions, prognosis, and patient outcomes. Due to its rarity, conducting larger multicenter studies across various countries is essential to validate these findings and investigate the underlying biological mechanisms in more detail.

## 4 Conclusion

The etiology of TMM remains unclear, yet certain conditions are deemed necessary for its development within recipient tumors. Despite its rarity, awareness of TMM among clinicians is crucial as the rising incidence of cancer may lead to more frequent cases. Small meningiomas in patients with known cancers should not be underestimated, as TMM can be the first presentation of a previously unknown cancer in over one-third of cases. While a preoperative diagnosis of meningioma containing metastasis may not alter surgical indications, it can influence the technical approach, prompting considerations for en bloc removal to prevent metastatic cell seeding. A multidisciplinary team involving neurosurgeons, pathologists, and oncologists is essential for timely diagnosis and accurate treatment planning. Further research is imperative to enhance our understanding and management of TMM.

## Author contributions

Conception and design: M.S, T.S, R.M,; acquisition of data: M.S, T.S, A.K.M.S, R.M; writing – original draft preparation: M.S, T.S, R.M; writing – reviewing and/or editing of manuscript: M.S, T.S, A.K.M.S, R.M. All authors have read and agreed to the published version of the manuscript.

## 5 Disclosure-conflict of interest statement

The authors whose names are listed below certify that they have no affiliations with or involvement in any organization or entity with any financial interest (such as honoraria; educational grants; participation in speakers’ bureaus; membership, employment, consultancies, stock ownership, or other equity interest; and expert testimony or patent-licensing arrangements), or non-financial interest (such as personal or professional relationships, affiliations, knowledge or beliefs) in the subject matter or materials discussed in this manuscript.

## Declaration of competing interest

The authors declare that they have no known competing financial interests or personal relationships that could have appeared to influence the work reported in this paper.
